# The Added Value of High Dose Spinal Cord Stimulation in Patients with Failed Back Surgery Syndrome after Conversion from Standard Spinal Cord Stimulation

**DOI:** 10.3390/jcm9103126

**Published:** 2020-09-27

**Authors:** Mats De Jaeger, Lisa Goudman, Koen Putman, Ann De Smedt, Philippe Rigoard, Wietse Geens, Maarten Moens

**Affiliations:** 1 Department of Neurosurgery, Universitair Ziekenhuis Brussel, Laarbeeklaan 101, 1090 Brussels, Belgium; matsdejaeger@gmail.com (M.D.J.); lisa.goudman@uzbrussel.be (L.G.); wietse.geens@vub.be (W.G.); 2 Pain in Motion International Research Group (PAIN), Department of Physiotherapy, Human Physiology and Anatomy, Faculty of Physical Education & Physiotherapy (KIMA),Vrije Universiteit Brussel (VUB), Laarbeeklaan 103, 1090 Brussels, Belgium; 3 Center for Neurosciences (C4N), Faculty of Medicine & Pharmacy, Vrije Universiteit Brussel (VUB), Laarbeeklaan 103, 1090 Brussels, Belgium; ann.desmedt@uzbrussel.be; 4 STIMULUS Consortium (reSearch and TeachIng neuroModULation Uz bruSsel), Universitair Ziekenhuis Brussel, Laarbeeklaan 101, 1090 Brussels, Belgium; 5 Department of Public Health (GEWE), Faculty of Medicine and Pharmacy, Vrije Universiteit Brussel (VUB), Laarbeeklaan 103, 1090 Brussels, Belgium; koen.putman@vub.be; 6 I-CHER, Interuniversity Center for Health Economics Research, Vrije Universiteit Brussel (VUB), Laarbeeklaan 103, 1090 Brussels, Belgium; 7 Department of Physical Medicine and Rehabilitation, Universitair Ziekenhuis Brussel, Laarbeeklaan 101, 1090 Brussels, Belgium; 8 Spine & Neuromodulation Functional Unit, Poitiers University Hospital, 86073 Poitiers, France; p.rigoard@chu-poitiers.fr; 9 Institut Prime, UPR CNRS 3346, ISAE-ENSMA, University of Poitiers, 86073 Poitiers, France; 10 PRISMATICS Lab (Predictive Research in Spine/Neuromodulation Management and Thoracic Innovation/Cardiac Surgery), Poitiers University Hospital, 86073 Poitiers, France; 11 Department of Radiology, Universitair Ziekenhuis Brussel, Laarbeeklaan 101, 1090 Brussels, Belgium

**Keywords:** failed back surgery syndrome, high-dose spinal cord stimulation, health-related quality of life, chronic pain management, EQ5D-3L, cohort study

## Abstract

Patients with Failed Back Surgery Syndrome (FBSS) report a considerably lower health- related quality of life (HRQoL), compared to the general population. Spinal cord stimulation (SCS) is an effective treatment to offer pain relief in those patients. Despite initial treatment success of SCS, its effect sometimes wears off over time. This study investigates the added value of high dose SCS (HD-SCS) in patients with unsatisfactory conventional SCS, from a quality of life perspective. Seventy-eight FBSS patients who were treated with conventional SCS that failed to provide pain relief, were recruited in 15 centers. HRQoL was assessed before converting to HD-SCS (baseline) and three times after converting to HD-SCS using the EuroQol-5D-3L. Quality adjusted life years (QALY) were calculated and compared with conventional SCS. An overall significant increase over time was seen in utility values of the EQ5D-3L, as the mean value at baseline 0.283 (±0.21) increased to 0.452 (±0.29) at 12 months of HD-SCS. This average increase in utility coincides with an average increase of 0.153 (±0.24) QALY’s in comparison to continued conventional SCS. Besides the potential of HD-SCS to salvage patients with failed responses to conventional SCS, this treatment seems to be a more efficient treatment than conventional SCS.

## 1. Introduction

Failed Back Surgery Syndrome (FBSS) is characterized by persisting back and/or leg pain of unknown origin either persisting despite surgical intervention or appearing after surgical intervention for spinal pain [1]. FBSS affects between 10 and 40% of patients following back surgery, resulting in more pain, increased opioid use, disability, and lower quality of life [2,3]. In comparison to other common chronic pain and medical conditions, the impact of FBSS on an individual’s health-related quality of life (HRQoL) and its socio-economic burden are considerably higher [4,5].

Spinal cord stimulation (SCS) is a minimal invasive treatment for neuropathic pain syndromes. It has not only shown to be effective in relieving pain, but also in improving disability and quality of life [6,7,8,9,10]. Due to both the increase in back surgeries with associated FBSS rates and mounting evidence of its effectiveness, SCS has found its way into routine clinical practice. Unfortunately, although successful SCS treatment may be a cost-effective therapeutic approach for FBSS patients compared to conventional medical management (CMM) [11], it is a rather expensive treatment because of high implantation costs, possible complications and annual maintenance costs [12]. In Belgium, around 900 SCS systems are implanted each year, with a yearly health insurance reimbursement cost of 12.5 million euros. About 9 million euros of these reimbursement costs are used for the materials of SCS, which makes this the highest cost driver in SCS treatment [13]. In addition to this price tag, conventional SCS has a significant long-term failure rate of approximately 30%. In 20–40% of patients, the initial effectiveness declines over time due to growing central nervous system tolerance [14,15]. In this case, the SCS system is often explanted and a substantial proportion of these explants happens before 2.25 years of SCS treatment [16,17], the so-called “break-even” point for SCS treatment when compared with CMM.

Alternative modes of SCS have been investigated during recent years, with as a main focus offering new treatment regiments for patients with failed conventional SCS, who lost their initial effectiveness over time [18,19]. High dose spinal cord stimulation (HD-SCS) is such a novel, subsensory SCS paradigm, based on pulse density rather than stimulating at a specific frequency. Studies have already showed that HD-SCS offers effective pain relief and higher patient satisfaction than conventional SCS [18,19]. Theoretically, prolonging the effectiveness of SCS treatment by conversion to HD-SCS after unsatisfactory conventional SCS could offer significant benefits not only from a clinical, but also from a health economical perspective. Successful continuation of SCS treatment by preventing system explants would increase the proportion of patients where the beneficial effect of pain reduction/relief remains at a lower cost over time.

It is our aim to investigate the effect of altering conventional to HD-SCS, as treatment for FBSS, from a health-related quality-of-life (HRQoL) perspective. Up till now, health economic evaluations that have found SCS to be cost-effective in comparison to CMM and re-operation have almost exclusively focused on “de novo patients”, thereby neglecting an important subgroup of FBSS patients which is the most difficult to treat [9,11,20,21]. Therefore, we investigated real-world data of HRQoL in a large FBSS study population, after failed or unsatisfactory conventional SCS [22].

## 2. Experimental Section

### 2.1. Study Population

“Rescue patients” were recruited between October 2016 and August 2018 in 15 Belgian neuromodulation centers. Rescue patients are defined as FBSS patients (defined as the surgical end-stage after one or several operative interventions on the lumbar neuroaxis, indicated to relieve lower back pain, radicular pain or the combination of both without positive effect [23]) with a numerical rating scale (NRS) score >3/10 for leg and/or back pain, treated with conventional SCS, and/or who were dissatisfied with conventional SCS as treatment. Patients were excluded if they had a life expectancy <1 year, a history of coagulation disorders, lupus erythematosus, diabetic neuropathy, rheumatoid arthritis, ankylosing spondylitis, an active malignancy and/or immune deficiency. Additionally, all patients were screened for technical failures and were excluded from study participation in case a technical issue was present.

### 2.2. Study Protocol

The study protocol was approved by the ethics committee of Universitair Ziekenhuis Brussel (B.U.N. 143201629180) and the local ethics committees of each participating center. The study protocol was registered on clinicaltrials.gov (NCT02787265). All patients provided written informed consent before enrolment. The study was conducted according to the revised Declaration of Helsinki (1998). Data are collected through the “High density in spinal cord stimulation: Virtual expert registry (DISCOVER)” registry [22]. This cohort study solely focuses on patients treated with conventional SCS with insufficient pain relief. Because of the unsatisfactory result of their conventional SCS, they were converted to HD-SCS. A baseline visit was scheduled just before converting patients to HD-SCS. After SCS conversion, three visits took place after respectively one month, three months and twelve months of HD-SCS. This means that all patients were previously implanted with SCS, more specifically with a RestoreSensor or PrimeAdanced implantable pulse generator (IPG) (Minneapolis, MN, USA), with unsatisfying results. Patients were converted to HD-SCS with a pulse density of 25% (500 Hz and 500 μs of pulse) in case of the RestoreSensor and 11.7% (450 Hz and 130 μs of pulse width) in case of a PrimeAdvanced IPG.

### 2.3. Health Outcome Measures

All participants completed a generic quality of life (QoL) questionnaire, the EQ-5D-3L [24]. The EQ-5D is the most widely used QoL questionnaire, developed by the EuroQol Group. It offers the advantage over disease-specific QoL-questionnaires, that its results can be compared across different health conditions and treatments. The EQ-5D-3L consists of 5 domains: mobility, self-care, pain/discomfort, usual activity and anxiety/depression. Each domain has 3 levels of severity, ranging from no problems to severe problems. Through a combination of the answers on each of those domains, patients are classified in 1 of 243 possible health states, where each health state is linked with an associated score, derived from a large sample of the population (0 = equivalent to death and 1 = equivalent to perfect health, negative values are possible). This scale of scores is referred to as the EQ5D “utility” score. Patients filled in an EQ-5D-3L at baseline, after 1 month, 3 months and 12 months of HD-SCS. Additionally, all patients filled in a Visual Analogue Scale (VAS) score whereby they rated their health from 0 to 100, where 0 is the worst health status they can imagine and 100 is the best health possible.

### 2.4. Statistical Analysis

Data analysis was performed using SPSS 24 (SPSS Inc., Chicago, IL, USA). Baseline patient characteristics were described by using absolute and/or relative frequencies, while continuous variables were summarized by mean values along with standard deviations (SD). The EQ-5D descriptive system was used to calculate utility scores by means of an algorithm that uses population-based Belgian value set. Quality-adjusted life-years (QALYs) were then calculated by multiplying the utility scores of the EQ-5D-3L by the time period (years) referring to the corresponding HRQOL, by using the area-under the curve (AUC). The QALY is a generic measure of disease burden, including both the quality and the quantity of life lived [25]. It is used in economic evaluations to assess the value of medical interventions. One QALY equates to one year in perfect health. We assumed that quality of life changed linearly between the assessments [26]. Health values at baseline (B) and after 12 months of HD-SCS (12M) were obtained through the time trade-off (TTO) approach [27,28]. The following equation was used to calculate AUC:

(1) $$AUC_{B-12M}\;=\left(\frac{\left(TTO_{B}+\;TTO_{1M}\;\right)}{2}\right)*\left(\frac{1}{12}\right)+\left(\frac{\left(TTO_{1M}+\;TTO_{3M}\;\right)}{2}\right)*\left(\frac{1}{6}\right)+\;\left(\frac{\left(TTO_{3M}+\;TTO_{12M}\;\right)}{2}\right)*\left(\frac{3}{4}\right)$$

If patients were not satisfied by their new stimulation paradigm and would like to return to their previous stimulation program, they were assumed to revert back to their original health state (linear decrease over time method). For patients lost to follow-up due to reasons non-related to their SCS paradigm, the last observation was carried forward until the last visit at 12 months [29]. Additionally, a complete case analysis was performed. Comparisons between enrollment and follow up were investigated using repeated measures analysis of variance (ANOVA) or a non-parametric equivalent (Friedman test) with time as within-subjects effect. Distribution of the data is assessed by the Shapiro-Wilk test.

## 3. Results

### 3.1. Patient Characteristics

A total of 78 patients with FBSS were enrolled in this study. Twenty-five males and 53 females participated with an average age of 56 ± 11.1 years. Table 1 presents an overview of the patient characteristics at baseline.

**Figure 1:**
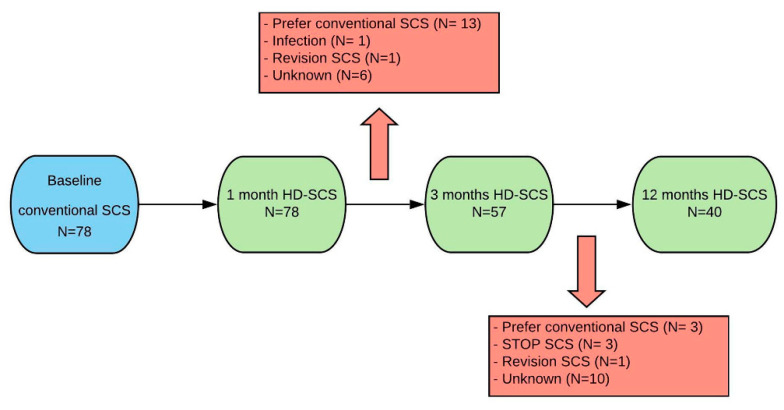
Flow chart of the study. Patients who received the status “unknown” are patients who missed their scheduled outpatient study visit. Abbreviations: HD-SCS: high dose Spinal Cord Stimulation, N: number of patients.

During the follow-up period 38 patients withdrew from the study for reasons specified in Figure 1. Patients who left the study due to unsatisfactory results with HD-SCS (*n* = 16) received their baseline utility score, as they were expected to return to their initial quality of life before the conversion to HD-SCS. Patients who did not continue with the trial for other reasons (*n* = 22) were corrected by means of last observation carried forward.

### 3.2. Health Related Quality of Life Outcome

At baseline, most patients reported problems in terms of pain, as 52 patients (66.7%) reported “extreme pain or discomfort” in this domain on the EQ-5D-3L. Furthermore, moderate problems with mobility and usual activities seemed to be the main issues for patients to be unsatisfied with their current conventional treatment (Table 2).

**Figure 2:**
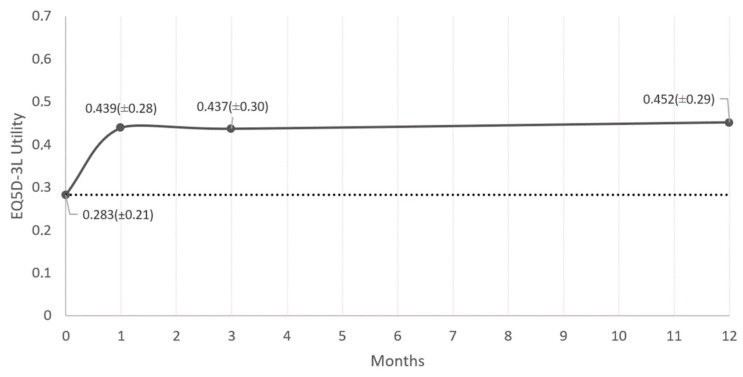
EQ-5D-3L mean utility values (±SD) plotted over time for HD-SCS (full line) and continued conventional SCS (dotted line) during the observational period. Abbreviations: HD-SCS: high dose Spinal Cord Stimulation, SCS: Spinal Cord Stimulation, SD: standard deviation.

The mean EQ-5D utility value increased from 0.283 at baseline to 0.452 at 12 months follow-up (Figure 2). Friedman test revealed an overall significant increase in utility values over time (*p* < 0.001), however a Wilcoxon signed ranks test showed only a significant increase between baseline and 1 month (Z = −4.71, *p* < 0.001) (Table 3). No further significant increases were found during the observational period.

The significant increase in utility values of the EQ-5D-3L is associated with an average increase in QALY of 0.153 (±0.24) after 12 months of HD-SCS. Twelve months after conversion from conventional to HD-SCS an increase in QALY was seen in 50 patients (64.1%), while 8 had no improvement and 20 were worse compared to baseline. A Friedman test revealed that there is no overall significant difference in VAS scores over time (χ2(3) = 5.919, *p* = 116), no post hoc analysis are performed due to the lack of an overall effect.

### 3.3. Complete Case Analysis

Out of the initial 78 patients, 40 patients completed the entire observational period. The health states in the group of patients with complete data did not differ much from the total population group, as most severe problems lie in the domain of “pain” (Table 4).

Of the initial 24 patients who reported severe problems because of pain, only eight still reported those problems after 12 months of HD-SCS. The mean EQ-5D utility value increased from 0.297 at baseline to 0.564 at 12 months follow-up (Table 5). A Friedman test revealed an overall significant increase in utility values over time (*p* < 0.001). A Wilcoxon signed ranks test showed only a significant increase between baseline and 1 month (Z = −4.86, *p* < 0.001). From all patients who completed follow-up, 35 out of 40 (87.5%) showed an increase in QALY after 12 months of HD-SCS, resulting in an average increase of 0.255(±0.26).

A Friedman test reveals that there is an overall statistically significant difference in VAS scores over time (χ2(3) = 12.217, *p* = 007), post hoc analysis shows a significant difference between baseline and 1 month (Z = −2.577, *p* = 010), between other time intervals no significant differences were found.

## 4. Discussion

Previous research has already shown that, in some cases, the effect of SCS wanes over time. These patients form a subgroup of SCS patients more difficult to treat, despite a permanent IPG implantation [18,30,31]. This particular group is mostly neglected in the field of neuromodulation studies where most research focuses on patients without prior exposure to SCS. However, it is precisely this group most in need for a solution to relieve their pain. With the emergence of new technologies and stimulation paradigms, already implanted IPGs may offer a solution for those for whom their original stimulation algorithm has lost effectiveness. This study is the first to evaluate HD-SCS after unsatisfactory conventional SCS in FBSS patients in a real-world context. By the design of this study, all patients were aware of the potential beneficial effects of conversion to HD-SCS. As such, the success of this conversion to HD-SCS will partly incorporate a placebo effect as well.

FBSS patients, prior to SCS implantation, report pain and limited capabilities as the greatest impairments of their quality of life. In a recent study by Scalone et al., 65% of FBSS patients reported on the EQ5D-3L extreme problems on the subdomain of “pain”, whereas 41.3% reported extreme problems on the subdomain of “usual activities” [32].

The population of rescue patients in this study (i.e., patients in whom the initial effectiveness of conventional SCS is lost over time), resembles the population of SCS virgin patients by reporting pain in 66.7% and impairment of usual activities in 33.3%. An important difference, however, is found in the domain of mobility, as “de novo” patients showed extreme problems in the domain of mobility. This is not the case in this study group of rescue patients, as most patients only reported moderate problems in this domain. After 12 months of HD-SCS, 20% reported extreme problems with pain and only 2.5% with usual activities on the EQ5D-3L.

When calculating the utility value of the EQ5D-3L, the baseline value of this specific subgroup is 0.283 (±0.21). This is a rather low value, considering that patients suffering from FBSS without previous treatment, revealed utility values ranging from 0.13 to 0.421 [4,5,33,34,35]. Another register-based study with pretreatment FBSS patients revealed utility values before SCS implantation of 0.22, showing little difference with our subgroup of rescue patients [33]. After twelve months of HD-SCS an increase in utility value of 0.17 is reported, which is considered a substantial improvement when we compare this value with values reported in the literature. Kemler et al. reported an increase of 0.22 after one year of SCS in combination with physiotherapy in a population of CRPS patients without previous SCS treatment [36]. Manca et al. on the other hand reported an increase in EQ5D utility values of 0.21 after six months of conventional SCS, compared to medical management, in new patients [34]. This improvement in utility values coincides with a QALY improvement of 0.153 (±0.24) after one year of conventional SCS. The best comparator to this study is a recent study by Zucco et al., in which an increase in QALY of 0.173 was found after 6 months of conventional SCS in 80 “de novo” FBSS patients with predominant leg pain [35].

An often-overlooked part of the EQ5D-3L is the descriptive component, depicted by a VAS where the patient is asked to score their general well-being on a scale of 0–100. In contrast to the utility values which are derived from the preferences of the general population, the EQ VAS generates data that represents what the patient’s own assessment of their health is. The EQ VAS of FBSS patients before salvation is 51.1 (±20.3) in this study. This is significantly higher than the novo SCS patients, as reported in the study of Scalone et al. with an EQ VAS of 37.4 [32]. EQ VAS comparison between our baseline results (51.1(±20.3)) and the results of Scalone et al. at 12 months of SCS (55) only indicates a small difference although patient satisfaction with SCS was highly incomparable.

A meta-analysis indicated that SCS results in a higher prevalence of chronic pain patients at work compared to before treatment [37]. Previously, it has been demonstrated within patients with burn injuries, that those who did not work were characterized by low HRQoL [38]. It might be possible that the gain in HRQoL in this study, resulted in an increased number of patients that is able to return to work. Therefore, future studies should take return to work into account to explore the full impact of HD-SCS in this population.

In the Discover registry, data was collected from 15 Belgian neuromodulation centers whereby patients with a NRS score >3/10 and/or who were dissatisfied with conventional SCS were eligible to participate. Eventually, 67.9% of the participants who took part in this study were females. This finding is in line with the reporting that chronic low back pain has a higher prevalence in females compared to males [39]. In 2019, a systematic review was conducted to evaluate the overall efficacy of SCS for patients with FBSS [40]. The study concluded that there was limited data on the effect of sex on SCS outcomes. Therefore, the authors believe that the higher proportion of females in this study is representative according to general prevalence rates, whereby the influence of sex on SCS outcomes is probably limited.

In this study, there was no need to schedule extra reprogramming visits, as most patients are already accustomed with their neurostimulator and were able to follow instructions after the initial reprogramming at baseline. No cost data were collected in the Discover registry. More research is needed to evaluate whether conversion to HD-SCS is also beneficial from a health-economical point of view.

Finally, this study did not systematically record the reason why patients were dissatisfied with conventional SCS before they were converted to HD-SCS. Future studies should also record this type of data to gain further insight in patient preferences regarding different stimulation types.

## 5. Conclusions

Real world data provide us with insights of the additional benefit offered by new stimulation paradigms, after unsatisfactory conventional SCS in FBSS patients from a HRQoL perspective. By converting from conventional SCS to HD-SCS, it is reasonable to assume that HD-SCS provides a prolongation or reignition of the initial effect of conventional SCS.

## Figures and Tables

**Table 1:** Patient characteristics at baseline.

Patient Characteristics at Baseline (*n* = 78)			
**Age (years)**			56 ± 11.1
**Sex**	Male		25 (32.1%)
	Female		53 (67.9%)
**Paresthesia coverage**	Back	Full	32 (41.0%)
		Partial	46 (59.0%)
	Leg	Full	41 (52.6%)
		Partial	37 (47.4%)
**Type of SCS**	RestoreSensor		7 (9.0%)
	PrimeAdvanced		71 (91.0%)
**NRS (/10)**	Low back pain		7.0 [5.0–9.0]
	Leg pain		7.0 [5.0–9.0]
**ODI**			52.8 ± 16.7
**PSQI**			12.3 ± 4.3
**EQ-5D-3L**			0.28 ± 0.21

Pain scores are expressed as median values with the first and third quartile. Mean values with standard deviations are reported for age, ODI, PSQI and EQ-5D-3L. For paresthesia coverage and type of SCS the exact counts with corresponding percentages are reported. Abbreviations: NRS: numerical rating scale, ODI: Oswestry disability index, PSQI: Pittsburgh sleep quality index, SCS: Spinal Cord Stimulation.

**Table 2:** Summary of reported problems in each domain of the EQ-5D-3L for all patients.

		Baseline	1 Month	3 Months	12 Months
**Domain**	**Problems**	*n* = 78	*n* = 78	*n* = 57	*n* = 40
**Mobility**	No	10 (12.8%)	17 (21.8%)	17 (29.8%)	15 (37.5%)
	Moderate	68 (87.2%)	61 (78.2%)	39 (68.4%)	25 (62.5%)
	Extreme	/	/	1 (1.8%)	/
**Selfcare**	No	36 (46.2%)	43 (55.1%)	35 (61.4%)	27 (67.5%)
	Moderate	39 (50.0%)	34 (43.6%)	21 (36.8%)	13 (32.5%)
	Extreme	3 (3.8%)	1 (1.3%)	1 (1.8%)	/
**Usual activities**	No	7 (9.0%)	17 (21.8%)	15 (26.3%)	15 (37.5%)
	Moderate	45 (57.7%)	51 (65.4%)	36 (63.2%)	24 (60.0%)
	Extreme	27 (33.3%)	10 (12.8%)	6 (10.5%)	1 (2.5%)
**Pain**	No	1 (1.3%)	6 (7.7%)	6 (10.5%)	9 (22.5%)
	Moderate	25 (32.1%)	40 (51.3%)	30 (52.6%)	23 (57.5%)
	Extreme	52 (66.7%)	32 (41.0%)	21 (36.8%)	8 (20.0%)
**Anxiety**	No	31 (39.7%)	41 (52.6%)	29 (50.9%)	24 (60.0%)
	Moderate	32 (41.0%)	25 (32.1%)	20 (35.1%)	13 (32.5%)
	Extreme	15 (19.2%)	12 (15.4%)	8 (14.0%)	3 (7.5%)

**Table 3:** Mean and median utility values (±SD; IQR) and VAS-score (0–100; ±SD; IQR) of the EQ-5D-3L of all patients receiving HD-SCS. Abbreviations: HD-SCS: high dose Spinal Cord Stimulation, IQR: interquartile range, SD: standard deviation, VAS: Visual Analogue Scale.

			Utility Values			
			Baseline	1 Month	3 Months	12 Months
**All patients (*n* = 78 with last observation carried forward for *n* = 22)**	**Utility**	**Mean**	0.283 (±0.21)	0.439 (±0.28)	0.437 (±0.30)	0.452 (±0.29)
		**Median**	0.236 [0.133–0.473]	0.551 [0.181–0.659]	0.473 [0.133–0.659]	0.515 [0.186–0.659]
	**VAS**	**Mean**	51.1 (±20.3)	53.4 (±20.7)	54.6 (±21.4)	54.9 (±20.6)
		**Median**	50.0 [40.0–65.0]	56.5 [38.5–70.0]	54.5 [37.0–70.0]	58.5 [40.0–70.0]

**Table 4:** Summary of reported problems in each domain of the EQ-5D-3L in patients with complete data.

		Baseline	1 Month	3 Months	12 Months
**Domain**	**Problems**	*n* = 40	*n* = 40	*n* = 40	*n* = 40
**Mobility**	*No*	7 (17.5%)	10 (25.0%)	14 (35.0%)	15 (37.5%)
	*Moderate*	33 (82.5%)	30 (75.0%)	26 (65.0%)	25 (62.5%)
	*Extreme*	/	/	/	/
**Self-care**	*No*	21 (52.5%)	26 (65.0%)	27 (67.5%)	27 (67.5%)
	*Moderate*	18 (45.0%)	14 (35.0%)	13 (32.5%)	13 (32.5%)
	*Extreme*	1 (2.5%)	/	/	/
**Usual activities**	*No*	5 (12.5%)	14 (35.0%)	14 (35.0%)	15 (37.5%)
	*Moderate*	21 (52.5%)	23 (57.5%)	23 (57.5%)	24 (60.0%)
	*Extreme*	14 (35.0%)	3 (7.5%)	3 (7.5%)	1 (2.5%)
**Pain**	*No*	1 (2.5%)	5 (12.5%)	6 (15.0%)	9 (22.5%)
	*Moderate*	15 (37.5%)	26 (65.0%)	26 (65.0%)	23 (57.5%)
	*Extreme*	24 (60.0%)	9 (22.5%)	8 (20.0%)	8 (20.0%)
**Anxiety**	*No*	16 (40.0%)	24 (60.0%)	23 (57.5%)	24 (60.0%)
	*Moderate*	15 (37.5%)	12 (30.0%)	11 (27.5%)	13 (32.5%)
	*Extreme*	9 (22.5%)	4 (10.0%)	6 (15.0%)	3 (7.5%)

**Table 5:** Mean and median utility values (±SD; IQR) and VAS-score (0–100; ±SD; IQR) of the EQ-5D-3L of patients with complete follow-up, receiving HD-SCS. Abbreviations: HD-SCS: high dose Spinal Cord Stimulation, IQR: interquartile range, SD: standard deviation, VAS: Visual Analogue Scale.

			Utility Values			
			Baseline	1 Month	3 Months	12 Months
**All patients (*n* = 40)**	**Utility**	**Mean**	0.297 (±0.22)	0.521 (±0.26)	0.542 (±0.30)	0.564 (±0.28)
		**Median**	0.236 [0.139–0.473]	0.644 [0.249–0.690]	0.618 [0.221–0.733]	0.577 [0.388–0.722]
	**VAS**	**Mean**	56.6 (±17.9)	62.6 (±15.5)	62.5 (±18.3)	62.6 (±16.6)
		**Median**	55.0 [49.0–70.0]	65.0 [50.0–71.0]	60.0 [50.0–80.0]	70.0 [50.0–70.0]
